# Modular Evolution and Population Variability of *Oikopleura dioica* Metallothioneins

**DOI:** 10.3389/fcell.2021.702688

**Published:** 2021-07-02

**Authors:** Sara Calatayud, Mario Garcia-Risco, Mercè Capdevila, Cristian Cañestro, Òscar Palacios, Ricard Albalat

**Affiliations:** ^1^Departament de Genètica, Microbiologia i Estadística, Facultat de Biologia, Institut de Recerca de la Biodiversitat (IRBio), Universitat de Barcelona, Barcelona, Spain; ^2^Departament de Química, Facultat de Ciències, Universitat Autònoma de Barcelona, Cerdanyola del Vallès, Spain

**Keywords:** appendicularian tunicate chordates, modular protein evolution, metallothionein domains, tandem domain repeats, population variants, intra-species variability

## Abstract

Chordate *Oikopleura dioica* probably is the fastest evolving metazoan reported so far, and thereby, a suitable system in which to explore the limits of evolutionary processes. For this reason, and in order to gain new insights on the evolution of protein modularity, we have investigated the organization, function and evolution of multi-modular metallothionein (MT) proteins in *O. dioica*. MTs are a heterogeneous group of modular proteins defined by their cysteine (C)-rich domains, which confer the capacity of coordinating different transition metal ions. *O. dioica* has two MTs, a bi-modular OdiMT1 consisting of two domains (t-12C and 12C), and a multi-modular OdiMT2 with six t-12C/12C repeats. By means of mass spectrometry and spectroscopy of metal-protein complexes, we have shown that the 12C domain is able to autonomously bind four divalent metal ions, although the t-12C/12C pair –as it is found in OdiMT1– is the optimized unit for divalent metal binding. We have also shown a direct relationship between the number of the t-12C/12C repeats and the metal-binding capacity of the MTs, which means a stepwise mode of functional and structural evolution for OdiMT2. Finally, after analyzing four different *O. dioica* populations worldwide distributed, we have detected several OdiMT2 variants with changes in their number of t-12C/12C domain repeats. This finding reveals that the number of repeats fluctuates between current *O. dioica* populations, which provides a new perspective on the evolution of domain repeat proteins.

## Introduction

*Oikopleura dioica* is a tunicate species of the appendicularian class in the chordate phylum. This species is emerging as a non-classical animal model in the field of evolutionary developmental biology (a.k.a. evo-devo) especially attractive for its unusually dynamic gene and genome evolution (reviewed in Ferrández-Roldán et al., 2019). At genome level, *O. dioica* has suffered numerous chromosomal rearrangements accompanied by a drastic process of compaction, becoming the smallest known chordate genome (Denoeud et al., 2010). At gene level, besides an extraordinary amount of gene duplications and losses, *O. dioica* sequences show high evolutionary rates, which are on average two-three times higher than in ascidians and vertebrates (Berna et al., 2012; Berna and Alvarez-Valin, 2014). *O. dioica* probably is the fastest evolving metazoan reported so far (Edvardsen et al., 2005; Denoeud et al., 2010). In addition, its pattern of amino acid substitution also shows some unusual traits in comparison with other chordates. Cysteines (Cys, C), for instance, are the less conserved amino acids in *O. dioica* proteins (Berna et al., 2012; Berná and Alvarez-Valin, 2015), whereas Cys are one of the most conserved amino acids according with classical analyses of protein evolution (Henikoff and Henikoff, 1992; Marino and Gladyshev, 2010). These exceptional evolutionary features make comparative studies between *O. dioica* and other chordate species suitable for understanding the functional and structural limits to which chordate genes and proteins can evolve.

In protein evolution, domains are considered evolutionary modules, and the majority of proteins of all living beings are multi-modular proteins that consist of several domains (Apic et al., 2001). While the creation of multi-modular proteins through shuffling of different domain types has been extensively analyzed (Apic et al., 2001; Björklund et al., 2005; Dohmen et al., 2020), the evolution of proteins made of tandem domain repeats is less understood (Björklund et al., 2006). To get new insights into the functional and structural evolution of these domain repeat proteins, we have focused on the multi-modular metallothioneins (MTs), using those of the fast evolving *O. dioica* species as case study.

Metallothioneins are proteins known for their high percentage of cysteines (Kojima et al., 1999), which confers them the capability of binding both essential and non-essential transition metals (reviewed in Capdevila et al., 2012; Blindauer, 2014). The Cys residues of MTs are arranged in distinctive motifs (i.e., CxC, CC, and CCC), whose number and distribution led to define different functional domains, originally for mammalian MTs (Braun et al., 1986), and later, in other animal MTs (Riek et al., 1999; Munoz et al., 2002; Baumann et al., 2017; Beil et al., 2019; Calatayud et al., 2021b). In chordates, for instance, vertebrate and cephalochordate MTs are bi-modular proteins with two domains that have diverse preferences and capacities for binding zinc (Zn), copper (Cu), or cadmium (Cd) ions (Capdevila and Atrian, 2011; Vasak and Meloni, 2011; Guirola et al., 2012; Artells et al., 2013). In contrast, most tunicate MTs are mono-modular proteins, whose single domain has a pervasive preference for Cd(II) ions (Calatayud et al., 2021a). The domain configuration of each MT is, indeed, functionally and structurally relevant because domains determine the formation of metal-thiolate clusters: domains with 9 Cys cluster with three divalent metal ions, while 11/12 Cys domains cluster with four divalent metal ions (e.g., mammalian β and α domains, respectively, Otvos and Armitage, 1980; Schultze et al., 1988). In addition, domain analyses have been shown to be helpful for elucidating the origin and evolutionary relationships of MTs in diverse groups of mollusks (Jenny et al., 2016; Nam and Kim, 2017; Calatayud et al., 2021b), and to reconstruct the complex evolutionary history of chordate MTs (Calatayud et al., 2021a).

*O. dioica* has two MTs, a bi-modular OdiMT1 and a multi-modular OdiMT2 (formerly OdMT1 and OdMT2) made of different number of domain repeats (Calatayud et al., 2018). The arrangement of Cys motifs in *O. dioica* domains diverges from that found in the MTs of other tunicates belonging to the ascidian and thaliacean classes, but it is similar to that of other appendicularian species of the same genus, *O. albicans* and *O. vanhoeffeni* (Calatayud et al., 2021a). Comparison of the appendicularian MTs show that the original *Oikopleura* MT domain had twelve cysteines (12C), and that this domain corresponds to previously described C7 + C5 subunits (Calatayud et al., 2018, 2021a). OdiMT1 would have therefore two 12C domains, but its amino-terminal domain was “trimmed” to become a t-12C domain that lacks the C5 subunit. OdiMT2 would be a multi-modular MT derived from an ancestral copy with a t-12C/12C domain organization, similar to the current OdiMT1, that was tandem duplicated five times yielding its domain repeat organization (Calatayud et al., 2018). In this work, we have pursued the analysis of *O. dioica* MTs, paying special attention to their modular configuration. First, we have characterized the metal-binding features of the original *Oikopleura* 12C domains –both the full-length (12C) and the trimmed (t-12C)–, revealing that although the 12C domain autonomously coordinates divalent metal ions, the t-12C/12C pair seems an improved form for divalent metal binding. Second, we have shown a direct relationship between the number of the t-12C/12C domain repeats and the metal-binding capacity of OdiMTs. Finally, taking advantage of the high level of genetic variation among *O. dioica* populations (Wang et al., 2020; Bliznina et al., 2021), we have detected population variants of OdiMT2 with changes in their number of t-12C/12C domains. Our data expose a high structural plasticity of MTs in *O. dioica* that, as if it was a natural test-bench, seems to be exploring the chordate limits of MT modularity.

## Materials and Methods

### Production and Purification of Recombinant Metal-MT Complexes

Production and purification of recombinant metal-MT complexes of proteins with different number and combinations of t-12C and 12C domains (see Table 1 for details; sequences of the domains are from Norwegian OdiMT1 and OdiMT2 sequences) was performed as described elsewhere (Calatayud et al., 2018, 2021b). In brief, synthetic cDNAs codifying the different constructs of 12-Cys domains were provided by Synbio Technologies (Monmouth Junction, NJ, United States), cloned in the pGEX-4T-1 expression vector (GE Healthcare, Chicago, IL, United States) and transformed in protease-deficient *E. coli* BL21 strain. Metal-MT complexes were produced in *E. coli* BL21 cultures expressing the recombinant plasmids, after induction with isopropyl-β-D-thiogalactopyranoside (100 μM) and supplementation with ZnCl_2_ (300 μM), CdCl_2_ (300 μM), or CuSO_4_ (500 μM). Metal-MT complexes were purified from the soluble protein fraction of sonicated bacteria by affinity purification of the GST-tagged proteins, and digestion with thrombin. Notice that the digestion with thrombin added two additional residues, Gly and Ser, at the N-terminal end of all purified proteins. These two amino acids do not interfere with the metal-binding features of recombinant MTs (Cols et al., 1997). The metal-MT complexes were concentrated with a 3 kDa Centripep Low Concentrator (Amicon, Merck), and fractionated on a Superdex-75 FPLC column (GE Healthcare) equilibrated with 20 mM Tris-HCl, pH 7.0 or with fresh 50 mM ammonium acetate, pH 7.0, and run at 0.8 mL min^–1^. The protein-containing fractions, identified by their absorbance at 254 nm, were pooled and stored at −80°C until use.

**TABLE 1:** Heterogously produced proteins containing different number and combinations of t-12C and 12C domains.

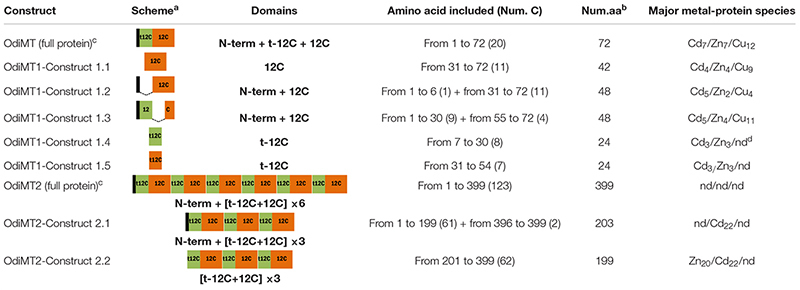

*^*a*^Colored boxes represents the six amino acids of the N-terminus (black), a “trimmed” 12C domain (green) and a complete 12C domain (orange).*

*^*b*^This number does not include two additional amino acids (Gly and Ser) added at the N-terminus of the recombinant proteins as consequence of the experimental design for the expression and purification of the metal-protein complexes (see section “Materials and Methods” for details).*

*^*c*^OdiMT1 and OdiMT2 full proteins were analyzed in Calatayud et al. (2018). ^*d*^nd, no detected.*

### Analysis of Metal-MT Complexes

All designed constructs of OdiMTs were characterized by means of mass spectrometry (ESI-MS) and spectroscopy (ICP-AES). An electrospray ionization mass spectrometry (ESI-MS) Micro Tof-Q Instrument (Brucker Daltonics Gmbh, Bremen, Germany) interfaced with a Series 1100 HPLC pump (Agilent Technologies) was used to determine the molecular mass of the recombinant proteins. The instrument was calibrated with ESI-L Low Concentration Tuning Mix (Agilent Technologies, United States) and the experimental conditions were set up as follows: injection of 10–20 μL of sample through a PEEK long tube (1–1.5 m × 0.18 mm i.d.) at 30–50 μL min^–1^; capillary-counterelectrode voltage, 3.5–5.0 kV; desolvation temperature, 90–110°C; dry gas, 6 L min^–1^. Data was acquired over an *m/z* range of 800–3,000. The liquid carriers were a 90:10 mixture of 15 mM ammonium acetate and acetonitrile at pH 7.0 and a 95:5 mixture of formic acid and acetonitrile at pH 2.4.

Element concentrations of S, Zn, Cd, and Cu in the sample were determined by Inductively Coupled Plasma Atomic Emission Spectroscopy (ICP-AES) by means of a Perkin-Elmer Optima 4300 DV (Waltham, United States) at the correct wavelength (S, 182.04 nm; Zn, 213.86 nm; Cd, 228.80 nm; Cu, 324.80 nm) under conventional conditions (Bongers et al., 1988). MTs concentration was calculated based on the S concentration obtained by ICP-AES, assuming that all the sulfur measured comes from peptides’ Cys and Met residues.

### Analysis of MT Variation

We analyzed the MT sequence variations of four geographically distant *O. dioica* populations: Norway, Japan (Osaka and Okinawa), Oregon, and Catalonia. We used Norwegian OdiMT1 (NCBI accession number CABV01001936.1) and OdiMT2 (NCBI accession number CABV01001042.1) sequences retrieved from the Oikobase genome database^1^ (Denoeud et al., 2010) as reference for blast searches in ANISEED^2^ and NCBI Sequence Read Archives^3^ for Japanese population (Osaka and Okinawa, respectively). Raw sequence data from SRA searches was assembled using SeqMan 8.0.2 (Pro Assembler) software from the DNASTAR Lasergene package, and manually inspected in order to reconstruct the MT sequences.

For Catalonian sequences, we PCR amplified the *MT* genes from specimens captured in the Mediterranean coast of Barcelona and cultured in our animal facility at the University of Barcelona (Martí-Solans et al., 2015). Primers and PCR conditions are listed in Supplementary Table 1. PCR products were cloned using Topo TA Cloning^®^ Kit of Invitrogen and sequenced at the Scientific and Technological Centers of the University of Barcelona, using the Big Dye Terminator v3.1 Cycle Sequencing Kit (Applied Biosystems) in an automatic sequencer (ABIPRISM 310, Applied Biosystems). For Oregonian sequences, we took advantage of a data of a genomic shotgun approach. All new MT sequences have been deposited in Genbank database at NCBI^4^.

For comparisons, MT sequences from different *O. dioica* populations were aligned with Aliview program (Larsson, 2014) and reviewed manually. Phylogenetic reconstructions were based on ML inferences calculated with PhyML v3.0 and automatic mode of selection of substitution model (Guindon et al., 2010) using protein sequence alignments. Sequences and the accession numbers used in this study are provided in Supplementary Table 2.

## Results and Discussion

### Functional Analysis of the Modularity in OdiMTs

#### Metal-Binding Capabilities of 12C and t-12C Domains

**FIGURE 1:**
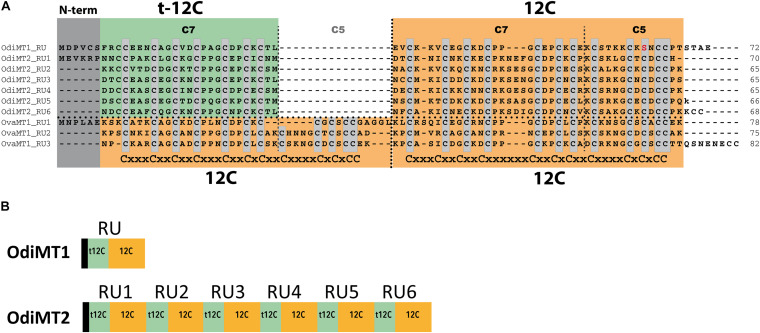
Domain organization of *O. dioica* metallothioneins. **(A)** Amino acid alignment of *O. dioica* OdiMT1 and OdiMT2 split in its repeat units (RU1–RU6). The sequence of *O. vanhoeffeni* MT (OvaMT1) split in its repeat units (RU1–RU3) has been included to show that the amino-terminal 12C domains of OdiMT1 and of the RU of OdiMT2 are “trimmed” (t-12C; green background), and lacks the C5 subunit. Conserved cysteines are highlighted in gray. The arrangement of Cys motifs of each 12C domain is showed below the alignment. Notice that the 12C domain (orange background) corresponds to previously described C7 + C5 subunits (Calatayud et al., 2018). **(B)** Schematic representation of the modular organization of OdiMT1 and OdiMT2 proteins. OdiMT1 consists of 6 amino acids of the N-terminus (black box) followed by a single repeat unit (RU) with a t-12C domain (green box) and a 12C domain (orange box), while OdiMT2 is made of six RU, RU1–RU6.

OdiMT1 is a 72 amino acid long protein (20 Cys, 28%) that can be divided in three parts: an N-terminal peptide made of six amino acids; next, an amino-terminal t-12C domain; and finally, a carboxyl-terminal 12C domain. OdiMT2 is a protein of 399 amino acids (123 Cys, 31%) that consists of the N-terminal peptide, and six tandem repeat units (RU1–RU6), each one made of a t-12C domain and a 12C domain (Figure 1; Calatayud et al., 2018). To investigate the functionality of this modular organization, we studied the formation of metal-MT complexes of recombinant proteins containing different combinations of t-12C and 12C domains (Table 1). The metal-binding capability of each construct was analyzed by inductively coupled plasma atomic emission spectroscopy (ICP-AES), and the species recovered identified by electrospray ionization mass spectrometry (ESI-MS) (Capdevila et al., 2012). ICP-AES was also used for metal-to-protein stoichiometry determination through the measurement of element composition of the samples (S, Zn, Cd, and Cu) (Bongers et al., 1988).

**FIGURE 2:**
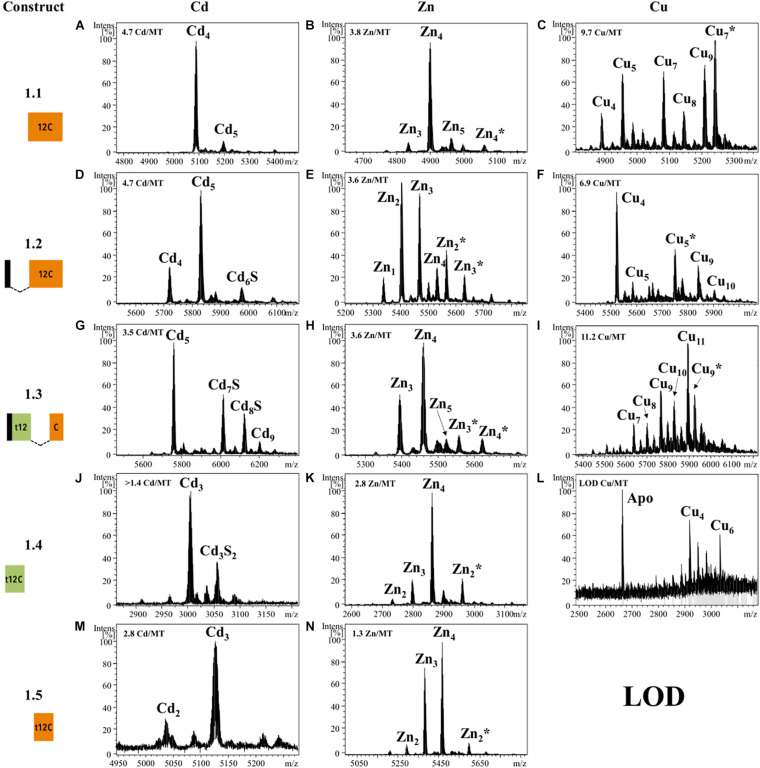
Deconvoluted ESI-MS spectra recorded at pH 7.0 of *OdiMT1* constructs 1.1 **(A–C)**, 1.2 **(D–F)**, 1.3 **(G–I)**, 1.4 **(J–L)**, and 1.5 **(M,N)** bioproduced with Cd **(A,D,G,J,M)**, Zn **(B,E,H,K,N)**, or Cu **(C,F,I,L)**. *Y*-axis represents the relative intensities of the peaks while the *X*-axis represents the m/z ratio equivalent to the MW (Da). The metal-to-protein ratio obtained by ICP-AES is included on the top left of each panel. It is indicated those cases in which the sample concentration was lower than the limit of detection (LOD). Glycosylated species are marked with an asterisk (*).

We first characterized the metal-binding features of the 12C domain by analyzing its ability to form metal complexes when expressed alone. Based on OdiMT1 sequence, we designed construct 1.1, which encoded a 42 amino acid peptide (residues from 31 to 72) comprising the 12C domain of OdiMT1 (Table 1). Notice that the 12C domain of OdiMT1 is, indeed, an 11C domain due to a Cys to Ser substitution in the carboxyl terminal region in comparison with prototypical 12C domain (thus, we named this domain as 11C/12C; Figure 1). Our results showed that this 11C/12C domain was able to yield almost unique Zn_4_- and Cd_4_-protein species (Figure 2), in a similar way that other domains with 11 or 12 Cys: the 11C mammal and echinoderm α domains (Stillman et al., 1987; Tomas et al., 2013), the 12C mollusk α domains (Digilio et al., 2009), the insect 12C MTs (Egli et al., 2006) and the ascidian and thaliacean 12C MTs (Calatayud et al., 2021a). These results revealed a significant structural and functional autonomy of *O. dioica* 11C/12C domain, which was able to form stable metal-protein clusters with Zn(II) and Cd(II). Regarding the metal preference of this domain, the mixture of multiple Cu_n_-protein complexes (n ranging from 4 to 10) in Cu(II) surplus productions (Figure 2) discarded a preference of this domain for monovalent Cu(I) ions. In addition, a preference for Cd(II) over Zn(II) might be indirectly inferred not only from the neatness of the ESI-MS spectra of the Cd-preparation but also from the fact that the domain rendered homometallic Cu(I) species in the Cu-preparations (Figure 2), which is characteristic of Cd-thioneins, whereas Zn-thioneins yield heterometallic Zn/Cu-MT complexes when expressed Cu-enriched media (Palacios et al., 2011). Overall, we concluded that the 11C/12C domain of OdiMTs formed stable clusters with four Cd(II) ions, which was in agreement with the biochemical features reported for the full OdiMT1 protein (Calatayud et al., 2018).

Although we obtained reliable results for the 11C/12C domain of OdiMT1, we wondered if the extra Cys found the N-terminal peptide of OdiMT1 could compensated the loss of one Cys in this domain, significantly improving its metal coordination features (i.e., enhancing specificity, increasing stability or metal-to-protein stoichiometries). To test this possibility, we designed two constructs: construct 1.2, which expressed a 48 amino acid peptide comprising the six N-term residues of OdiMT1 (which included the extra Cys) fused to the 11C/12C domain (Table 1); and construct 1.3, which also expressed a 48 amino acid peptide comprising the six N-term residues fused to a 12C domain resulting from the combination of the t-12C domain of OdiMT1 (from 7 to 30) with the 18 last residues of the carboxyl-terminus of the protein (from 55 to 72) (Table 1). Although both constructs rendered Cd_5_-protein complexes as major species according to ESI-MS data (Figure 2), the samples recovered from both, Zn- and Cd-supplemented cultures, resulted to be a significant mixture of metal-protein complexes and some Cd-protein species contained sulfide labile ligands (Figure 2). The presence of S^2–^ ions is probably due to the incapability of these artificially designed peptides to build a stable metal cluster as these “extra” ligands can aid in the stabilization of the metal clusters (Capdevila et al., 2005). This, together with the heterogeneity of the samples suggested, therefore, that the 1.2 and 1.3 constructs have not improved the metal binding abilities of construct 1.1, questioning the contribution of the extra Cys in the N-terminal peptide to the metal coordination, and reinforcing the functional entity of the 11C/12C domain as an efficient solution for coordinating divalent metal ions emerged during the evolution of the *Oikopleura* lineage.

Next, we investigated the functionality of the t-12C domain, which is a 12C domain lacking the carboxyl C5 subunit, and thereby, containing only 7/8 cysteines. We analyzed the metal-binding features of two different t-12C domains expressed by two constructs. Construct 1.4 produced the t-12C domain of OdiMT1, from residues 7 to 30. Construct 1.5 produced the t-12C domain resulting from the truncation of the last 18 residues of the 11/12C domain of OdiMT1, from residues 31 to 54 (Table 1). Our analyses showed that both constructs mainly bind three divalent metal ions, either Zn(II) or Cd(II) (Figure 2). This metal-to-protein stoichiometry agreed with the possibility that the seven divalent metal ions (M_7_) coordinated by the full OdiMT1 protein are organized in two metal clusters: an 11C/12C domain cluster with four metal ions (M^*II*^_4_) at the carboxyl-end of the MT, and a t-12C domain cluster with three metal ions (M^*II*^_3_) at the amino-term region. The t-12C domains rendered, however, multiple metallospecies and some Cd-protein species with sulfide ligands (Figure 2), which suggested that the t-12C domain expressed alone did not efficiently bind the metals by itself, and that it would require the neighboring 11C/12C domain to properly coordinate the seven metal ions in the full MT protein (Calatayud et al., 2018).

#### Metal-Binding Capabilities of t-12C/12C Repeats

**FIGURE 3:**
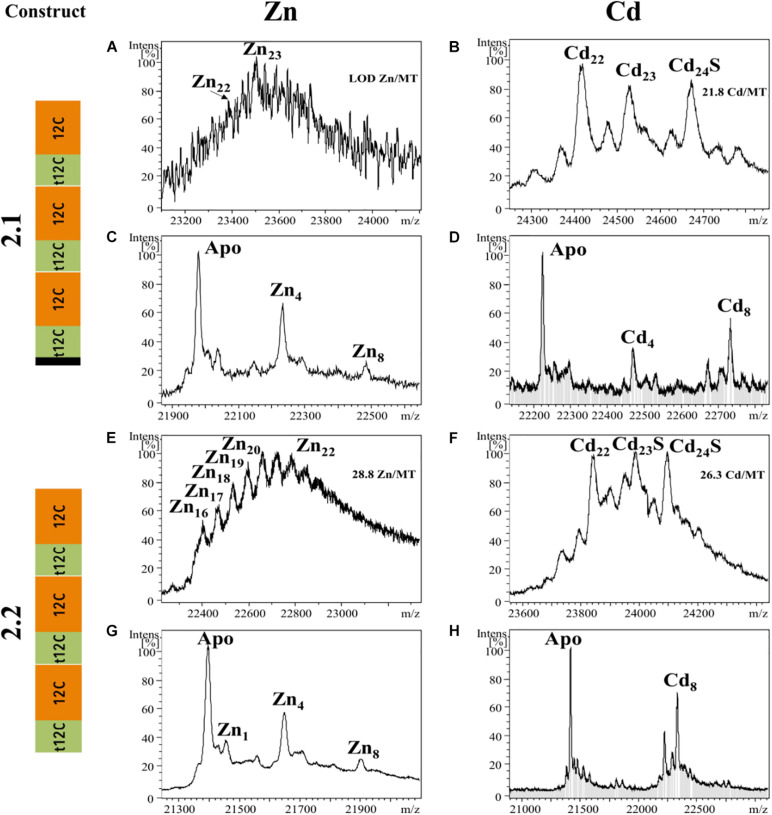
Deconvoluted ESI-MS spectra recorded at pH 7.0 **(A,B,E,F)** and pH 2.4 **(C,D,G,H)** of *OdiMT2* constructs 2.1 **(A–D)** and 2.2 **(E–H)** bioproduced with Zn **(A,C,E,G)** or Cd **(B,D,F,H)**. *Y*-axis represents the relative intensities of the peaks while the *X*-axis represents the m/z ratio equivalent to the MW (Da). The metal-to-protein ratio obtained by ICP-AES is included on the top right of panels **(A,B,E,F)**. It is indicated those cases in which the sample concentration was lower than the limit of detection (LOD).

We also analyzed the metal-binding features of tandem repeats of t-12C/12C domains as they are found in OdiMT2. We designed two constructs. Construct 2.1, encoding a 203 amino acid protein, comprised the three amino-terminal RU (i.e., RU_1_RU_2_RU_3_; residues 1–199), plus the four last amino acids (two of them Cys) of the carboxyl-end (from 396 to 399). Construct 2.2, encoding a 199 amino acid protein, comprised the three carboxyl-terminal RU (i.e., RU_4_RU_5_RU_6_) plus the four last amino acids (from 201 to 399) (Figure 1 and Table 1). Recombinant synthesis of the two partial OdiMT2 proteins –RU_1_RU_2_RU_3_ and RU_4_RU_5_RU_6_– in Zn(II)- and Cd(II)-enriched *E. coli* cultures rendered a low yield of metal-protein complexes, but ICP-AES analyses showed that both produced complexes with divalent metal ions, mostly with Cd(II) ions. ESI-MS analyses (Figure 3) showed mixtures of metal-protein complexes with different stoichiometries, and Cd-protein complexes with sulfide ligands, being Zn_2__0__–__2__1_, Cd_2__2__–__2__3_, and Cd_2__3__–__24_S-complexes the major species (Figure 3). The metal-to-protein stoichiometry of these species was close to what was expected considering that the partial proteins consist of three RU, each one equivalent to a full-length OdiMT1 that binds seven Zn(II)/Cd(II) ions, i.e., 3 RU × 7 M^*II*^ = 21 total divalent metal ions. The two additional Cys at the carboxyl-end, the presence of sulfide ligands, and the fact that multiple metallospecies are common in multi-modular MT productions (Palacios et al., 2014; Iturbe-Espinoza et al., 2016) could account for the slightly high metal content (up to 24 ions) observed in some of them. Interestingly, ESI-MS spectra of both constructs at pH 2.4 showed species loaded with 4 and 8 divalent metal ions (Figure 3). These species might be reflecting the t-12C/12C organization of each RU, in which the “standard” 12C domain would be more reluctant to release its four metal ions than the “trimmed” t-C12 domain. Overall, the metal stoichiometry of the partial MTs pointed to a direct relationship between the number of the t-12C/12C repeats and the metal-binding capacity of multi-modular OdiMTs.

In conclusion, our analyses suggested that during the evolution of MTs in *O. dioica*, an ancestral 12C domain was tandem duplicated. The N-terminal 12C copy was trimmed (t-12C domain), partially losing its autonomy for metal binding, and the t-12C/12C pair became the optimized functional unit. Afterward, this primeval t-12C/12C *OdiMT* gene was duplicated. While one of the duplicates remained unaltered as the current *OdiMT1*, the other copy suffered several internal tandem duplications of the functional t-12C/12C pair in an evolutionary process that stepwise changed the number of domain repeats affecting the metal binding capacity of the new multi-modular OdiMT2. Domain expansions that generate high metal-binding capacity MTs such as OdiMT2, gene duplications that lead to the amplification of the number of *MT* genes such as those of *CUP1* in yeast (Adamo et al., 2012), and mutations in regulatory regions that lead to the overexpression of MTs in insects (Costa et al., 2012; Catalan et al., 2016) appear to be different ways of increasing the physiological capabilities of the organisms to adapt to diverse conditions of metal bioavailability and other environmental stress situations.

### Genetic Variation in *O. dioica* MTs

**FIGURE 4:**
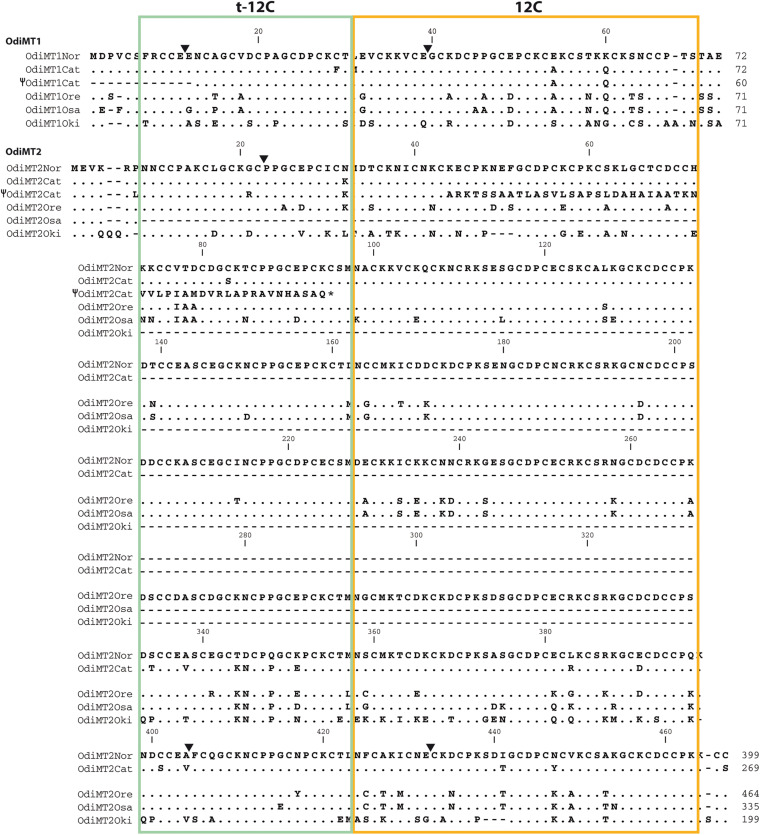
Amino acid alignments of OdiMT1 and OdiMT2 variants form Norwegian (NOR), Catalonian (CAT), Oregonian (ORE), Osaka (OSA), and Okinawa (OKI) *O. dioica* populations. Norwegian sequences are used as reference; dots and dashes indicate amino acid identity and gaps, respectively. Black arrowheads indicate the intron positions relative to the amino acid sequences. The sequences of two hypothetical OdiMT pseudogenes –i.e., Ψ OdiMT1_C__AT_, lacking the first 12 residues, and Ψ OdiMT2_C__AT_, with a premature stop codon (*)– are also included. Trimmed 12-Cys domains (green box) and full-length 12-Cys domains (orange box) at the N- and C-terminal regions of the RU, respectively, are depicted.

The peculiar structural and evolutionary characteristics of OdiMTs together with the high evolutionary rate of *O. dioica* (Denoeud et al., 2010; Berna et al., 2012; Berna and Alvarez-Valin, 2014), and significant level of sequence variation detected between *O. dioica* populations (Wang et al., 2015, 2020; Bliznina et al., 2021), prompted us to investigate the OdiMT sequences in several *O. dioica* populations worldwide distributed. We analyzed the MT sequences from animals from Norway (NOR), Oregon (ORE), Japan (Japanese specimens were from two different localizations, Osaka (OSA) and Okinawa (OKI), and we have analyzed them separately), and Catalonia (CAT), representing four geographically distant *O. dioica* populations: north Atlantic, eastern Pacific, western Pacific and Mediterranean populations, respectively. We used as guiding reference the Norwegian sequences (*OdiMT*_NOR_) (Figure 4 and Supplementary Figure 1) because they were the first sequences identified (Calatayud et al., 2018).

Both *OdiMT1* and *OdiMT2* genes were present in all analyzed populations (Figure 3), but comparison among populations revealed important differences affecting three aspect: (i) sequence variability, (ii) presence of non-functional allelic variants, and (iii) differences in the number of RU in the case of OdiMT2. Regarding sequence variability, protein sequence comparisons reveal amino-acid identities ranging from 94.4% between Norway and Barcelona, up to 63.9% when compared with the sequence of Okinawa, which appeared as the more distant population to any other one (Table 2). In contrast to the overall sequence variation –82.6 and 87.7% of average amino acid identity (excluding the divergent Okinawa sequences) for OdiMT1 and OdiMT2, respectively, Cys residues were nearly invariants, with 98.3% preservation. This high Cys conservation contrasted with the fact that Cys are the less conserved amino acids in *O. dioica* proteins (Berna et al., 2012; Berná and Alvarez-Valin, 2015), suggesting that Cys substitutions are negatively selected due to the functional restrictions imposed by metal coordination. Moreover, the fact that amino acid identities persistently were slightly lower than nucleotide identities (Table 2) indicated that nucleotide substitutions are significantly affecting non-synonymous positions, which can be considered an indication that MT variability among populations might probably be under positive selection.

**TABLE 2:** Percentage identity^a^ of nucleotide (above diagonal) and amino acid sequences (below diagonal) of the OdiMT coding regions from five different *O. dioica* populations, Norway (NOR), Catalonia (CAT), Osaka (OSA), Okinawa (OKI), and Oregon (ORE).

	OdiMT1_N__OR_	OdiMT1_C__AT_	OdiMT1_O__SA_	OdiMT1_O__KI_	OdiMT1_O__RE_
OdiMT1_N__OR_	–	97.26	84.02	73.87	83.56
OdiMT1_C__AT_	94.44	–	84.47	72.97	83.56
OdiMT1_O__SA_	76.38	76.38	–	70.58	93.52
OdiMT1_O__KI_	67.12	64.32	63.88	–	69.09
OdiMT1_O__RE_	79.16	79.16	90.27	66.66	–

	**OdiMT2_N__OR_**	**OdiMT2_C__AT_**	**OdiMT2_O__SA_**	**OdiMT2_O__KI_**	**OdiMT2_O__RE_**

OdiMT2_N__OR_	–	96.42	86.62	70.40	86.68
OdiMT2_C__AT_	94.07	–	85.81	70.72	87.42
OdiMT2_O__SA_	85.42	81.16	–	73.95	92.77
OdiMT2_O__KI_	67.79	68.75	67.59	–	73.76
OdiMT2_O__RE_	87.00	86.67	91.69	69.23	–

**^*a*^For the calculation of sequence identities, a gap between two sequences has been considered as a single difference.**

Noteworthy, during the identification of the Catalonian MTs, we detected some non-functional allelic variants of *OdiMT1* and *OdiMT2* genes. One variant displayed a 35 nucleotide (nt) deletion at the 5’-end of the *OdiMT1* genomic region that eliminated the first exon, and therefore likely resulted in a pseudogene, Ψ*-OdiMT1_*C*__*AT*_* (Figure 4 and Supplementary Figure 1). In another variant, we found a 7 nt deletion at the beginning of the exon 2 of the *OdiMT2* that caused a frame-shift mutation that resulted in a change of the amino acid sequence and a premature stop codon (Figure 4 and Supplementary Figure 1). The functionality of the truncated protein codified by Ψ*-OdiMT2_*C*__*AT*_* was unlikely because only 44% (41 out of 92) of the amino acids were similar to the OdiMT2 sequence (Figure 4). The presence of these non-functional alleles for both *MTs*,Ψ*-OdiMT1_*C*__*AT*_*, and Ψ*-OdiMT2_*C*__*AT*_*, in the Mediterranean population opened the possibility that some *O. dioica* specimens might lack functional MTs. Further analyses of the presence and frequencies of non-functional variants in different populations could reveal different selective pressures related to variations in heavy-metal amounts of different environments.

**FIGURE 5:**
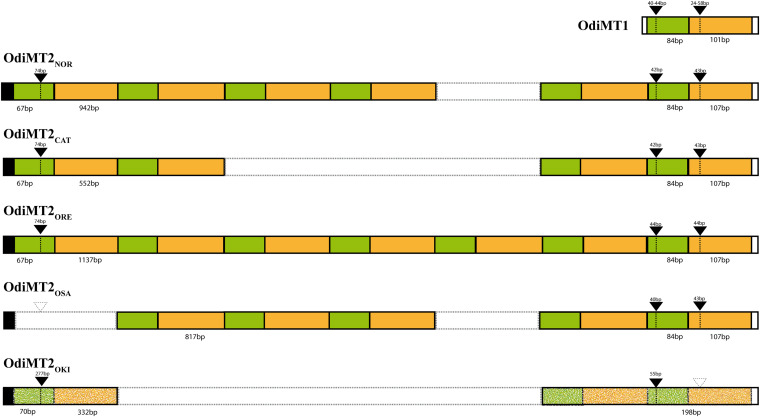
Schematic representation of the organization of RU in OdiMTs. The t-12C/12C RU in OdiMT1s (top) and in OdiMT2 variants with six (OdiMT2_N__OR_), four (OdiMT2_C__AT_), seven (OdiMT2_O__RE_), five (OdiMT2_O__SA_), and three (OdiMT2_O__KI_) RU are depicted following the color code of Figure 1. Repeats are positioned on the basis of intron positions (black arrowheads; dashed arrowheads denote absent introns) and phylogenetic analysis (Supplementary Figure 2). Exon and intron sizes are indicated. The high divergence of the RU of OdiMT2_O__KI_ is highlighted by the dotted pattern.

The most conspicuous difference of *OdiMT* genes between *O. dioica* populations was the identification of *OdiMT2* encoding proteins with variable number of RUs (i.e., t-12C/12 pairs) (Figures 4, 5). OdiMT2_O__RE_ with seven repeats (RU_1_–RU_7_) was the longest one, followed by OdiMT2_N__OR_, with six repeats, OdiMT2_O__SA_, with five repeats, OdiMT2_C__AT_, with four repeats, and OdiMT2_O__KI_, with three repeats. The conservation in the *OdiMT2* alleles of the introns both at the 5′- and 3′-ends of the gene (Figure 5), together with phylogenetic reconstructions based on the sequence alignments of the repeats (Supplementary Figure 2), suggested that the increase/decrease in the number of repeats was the result of internal tandem duplications/losses due to recombination events. Phylogenetic analysis suggested that some expansions of the RU preceded the divergence of populations, while some independent gains or losses also occurred (Supplementary Figure 2). In OdiMT2_O__KI_, for instance, duplications of RU seemed to have taken place independently form other OdiMT2s, suggesting again that this population is the most divergent among all populations. Finally, the fact that in all variants the repeat units comprised complete t-12C/12C pairs supported a modular, step-wise evolution for OdiMTs, and agreed with the functional analysis of the domains that demonstrated that despite the autonomy of the 12C domain, the t-12C/12C pair appeared as an improved functional unit for divalent metal binding (see section “Functional Analysis of the Modularity in OdiMTs”).

Proteins with domain repeats have been observed to be particularly common in multicellular species (Apic et al., 2001), especially in vertebrates (Björklund et al., 2006). The exact mechanism for repeat expansion remains to be discovered, but evidence of the expansion of repeats come from the fact that orthologous proteins might have different numbers of domain repeats in different species (Björklund et al., 2006). The number of domain repeats is, however, well conserved within a species, and it does not present intraspecific variability in terms of repeat unit gains/losses (Schaper et al., 2014). The population variability of OdiMT2s here exposed is, therefore, surprising, and *O. dioica* challenges again the standard patterns of gene and protein evolution, opening a new perspective in the functional and structural evolution of domain repeat proteins.

In summary, our results suggested that the modular organization provides MTs with a high structural and functional plasticity and dynamism, as it demonstrates the detection of variants with variable number of t-12C/12C repeats. These features seem to have facilitated the creation of large multi-modular MTs with high cysteine content and a high capacity of metal binding. Large multi-modular MTs have been described in other organisms (Palacios et al., 2014; Iturbe-Espinoza et al., 2016; Dallinger et al., 2020; Calatayud et al.,2021a,b), some of them associated with biological adaptations (Palacios et al., 2014; Iturbe-Espinoza et al., 2016; Jenny et al., 2016; Baumann et al., 2017). These large MTs did not show, however, the complexity level reached by *O. dioica* proteins, nor the inter-population variability described here. It seems, therefore, that *O. dioica* would be exploring the limits of chordate MT evolvability since, although we still do not know if adaptive selection to different environmental conditions would be driving the changes in the number of the t-12C/12C repeats, the more genetic variation there is, the greater the capacity for adaptive evolution of a biological system.

## Data Availability Statement

The datasets presented in this study can be found in online repositories. The names of the repository/repositories and accession number(s) can be found in the article/ Supplementary Material.

## Author Contributions

RA was responsible for the project coordination. RA, CC, ÒP, and MC conceived and designed the experiments. SC, CC, and RA collected the MT sequences from databases and elaborated the evolutionary inferences. SC performed the cloning and recombinant synthesis of the analyzed proteins. MG-R performed the ICP-AES, CD, UV-vis, and ESI-MS experiments. MG-R, ÒP, and MC analyzed the metal-binding data. SC and MG-R were responsible of the figures and tables. All authors discussed the experimental results, were responsible for writing the manuscript, commented on the manuscript, and agreed to its final version.

## Conflict of Interest

The authors declare that the research was conducted in the absence of any commercial or financial relationships that could be construed as a potential conflict of interest.

## Footnotes

1 http://oikoarrays.biology.uiowa.edu/Oiko

2 https://www.aniseed.cnrs.fr/

3 https://www.ncbi.nlm.nih.gov/sra/

4 https://www.ncbi.nlm.nih.gov/genbank/
