# Supplementary material for: Modular Evolution and Population Variability of Oikopleura dioica Metallothioneins
Source: Front Cell Dev Biol. 2021 Jul 2;9:702688. doi: 10.3389/fcell.2021.702688 (PMC8283569; doi:10.3389/fcell.2021.702688)
Supplement: Supplementary file 1 [file Data_Sheet_1.PDF]

Supplementary Figure 1

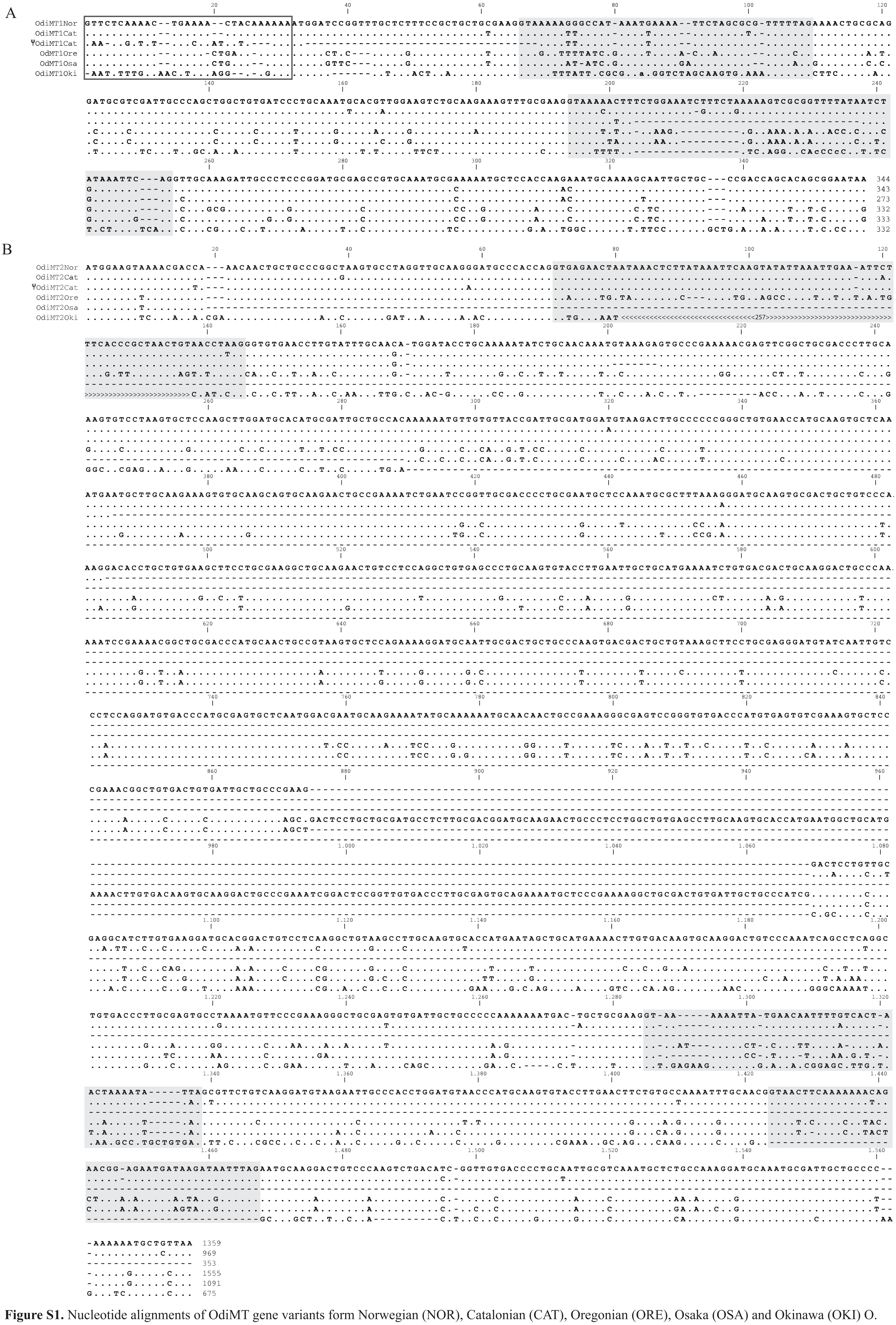

Figure S1. Nucleotide alignments of OdiMT gene variants form Norwegian (NOR), Catalonian (CAT), Oregonian (ORE), Osaka (OSA) and Okinawa (OKI) O. dioica populations. Norwegian sequences used as reference; dots and dashes indicate nucleotide identity and gaps, respectively. Grey boxes indicate introns relative to the genomic sequence alignment. (A) OdiMT1 genes. Black box depicts the sequence upstream of the ATG, revealing that a 35 nt deletion at the 5'-end of the Ψ-OdiMT1CAT removed the first exon. (B). OdiMT2 genes. Arrows in first intron of OdiMT2OKI indicate that the full nucleotide sequence is not included because the large size of the intron.
