# Supplementary material for: Modular Evolution and Population Variability of Oikopleura dioica Metallothioneins
Source: Front Cell Dev Biol. 2021 Jul 2;9:702688. doi: 10.3389/fcell.2021.702688 (PMC8283569; doi:10.3389/fcell.2021.702688)
Supplement: Supplementary file 2 [file Data_Sheet_2.PDF]

Supplementary Figure 2.

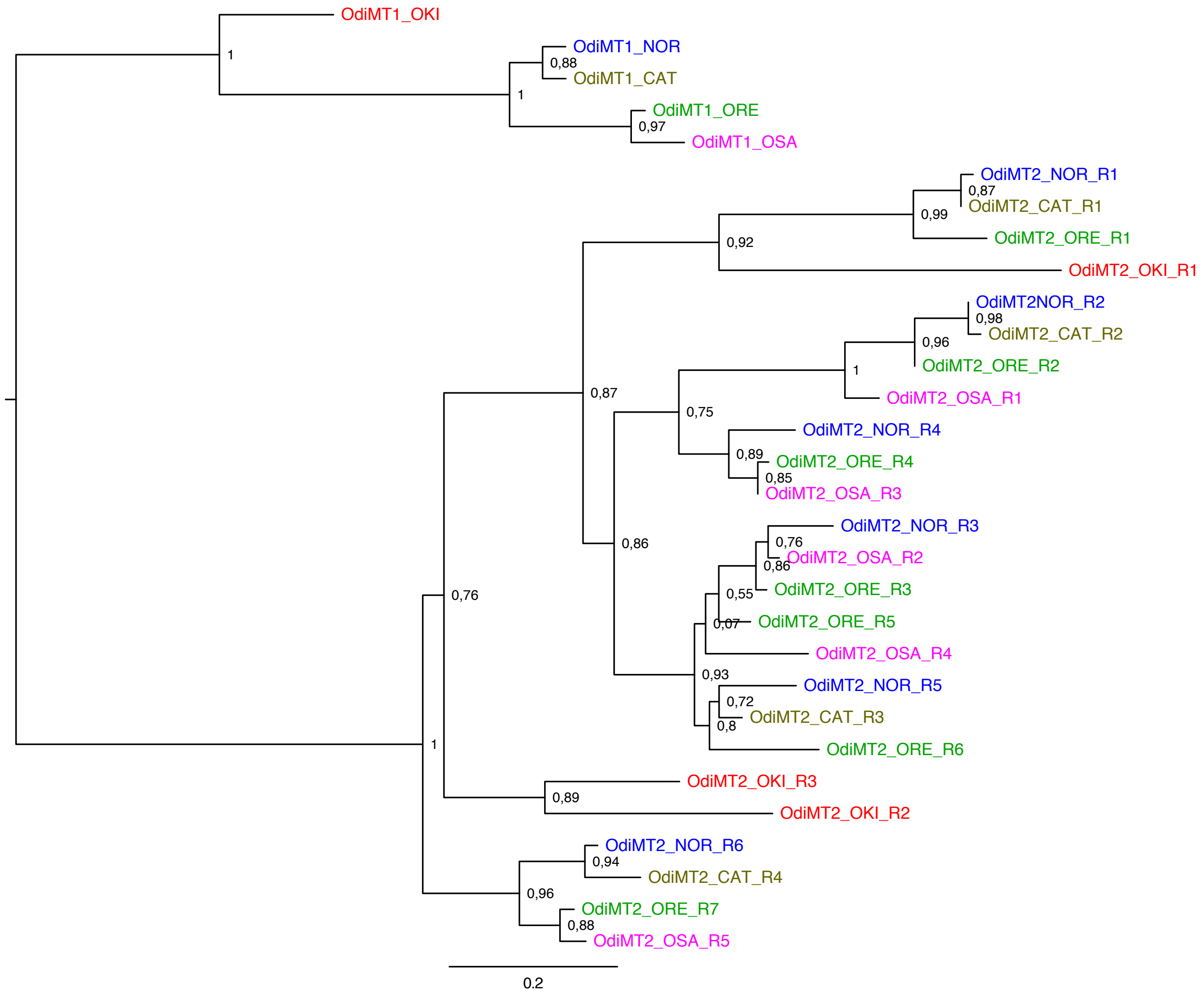

Supplementary Figure 2. Phylogenetic tree of the RU of OdiMT1 and OdiMT2 proteins from Norwegian (NOR; blue), Catalonian (CAT; olive), Oregonian (ORE; green), Osaka (OSA; purple) and Okinawa (OKI; red) *O. dioica* populations. RU are numbered from the amino- to the carboxyl-end of each MT variant as represented in **Figure 5**. Values for the approximate likelihood ratio test (aLRT) are shown at nodes The scale bar indicates amino acid substitutions.
