# Supplementary material for: Modular Evolution and Population Variability of Oikopleura dioica Metallothioneins
Source: Front Cell Dev Biol. 2021 Jul 2;9:702688. doi: 10.3389/fcell.2021.702688 (PMC8283569; doi:10.3389/fcell.2021.702688)
Supplement: Supplementary file 3 [file Table_1.pdf]

Table S1. Primers used for *OdiMT1* $_{CAT}$ and *OdiMT2* $_{CAT}$ *PCR-amplification and PCR conditions.*

Gene	Forward	Reverse	PCR conditions
OdiMT1$_{CAT}$	5' GGGGAATCGATATTGACAAATCTTCAA 3'	5' GGGCTCGAGTTATTCCGCTGTGCTGGTCGGGCAG 3'	94°C 3 min; 94°C 20 sec, 55ªC 30 sec and 72°C 1.30 min x40 cycles; 72°C 10 min
OdiMT2$_{CAT}$	5' GGGGGATCCATGGAAGTAAAACGACC 3'	5' GGTTCAGACGAAATTTGTCCCG 3'	98°C 30sec; 98°C 10 sec, 60°c 30 sec and 72°C 3 min x35 cycles; 72°C 10 min
