# Supplementary material for: Modular Evolution and Population Variability of Oikopleura dioica Metallothioneins
Source: Front Cell Dev Biol. 2021 Jul 2;9:702688. doi: 10.3389/fcell.2021.702688 (PMC8283569; doi:10.3389/fcell.2021.702688)
Supplement: Supplementary file 4 [file Table_2.pdf]

Table S2. Accession numbers and protein sequence of OdiMTs from different *O. dioica* populations.

OdiMT1

OdiMT1$_{NOR}$	CABV01001936.1	MDPVCSFRCCEENCAGCVDCPAGCDPCKCTLEVCKKVCEGCKDCPPGCEPCKCEKCSTKKCKSNCCPTSTAE
OdiMT1$_{CAT}$	MH547307	MDPVCSFRCCEENCAGCVDCPAGCDPCKFTMEVCKKVCEGCKDCPPGCEPCKCAKCSTKQCKSNCCPTSTAE
ΨOdiMT1$_{CAT}$	MH577048	NCAGCVDCPAGCDPCKCTLEVCKKVCEGCKDCPPGCEPCKCAKCSTKQCKSNCCPTSTAE
OdiMT1$_{ORE}$	MH577047	MDSCSFRCCEENCTGCADCPAGCDPCKCTLGVCKKVCEGCADCPAGCDPCKCAKCSNKQCKTSCCPTSSSE
OdiMT1$_{OKI}$	SRA Project	MDCSTRCCEASCEGCVSCPPGCDPCKCSLDSCKKVCQGCRDCPPGCDPCKCSKCSANGCKCSCCAATNTSA
OdiMT1$_{OSA}$	SRA Project	MEFCSFRCCEGNCPGCADCPAGCDPCKCTLGVCKKVCEGCKDCAAGCDPCKCAKCSNKQCKTSCCPTSSSE

OdiMT2

OdiMT2$_{NOR}$	CABV01001042.1	MEVKRPNNCCPAKCLGCKGCPPGCEPCICNMDTCKNICNKCKECPKNEFGCDPCKCPKCSKLGCTCDCCHKKCCVTDCDG<br>CKTCPPGCEPCKCSMNACKKVCKQCKNCRKSESGCDPCECSKCALKGCKCDCCPKDTCCEASCEGCKNCPPGCEPCKCTLN<br>CCMKICDDCKDCPKSENGCDPCNCRKCSRKGCNCDCCPSDDCCKASCEGCINCPPGCDPCECSMDECKKICKKCNNCRKG<br>ESGCDPCECRKCSRNGCDCDCCPKDSCCEASCEGCTDCPQGCKPCKCTMNSCMKTCDKCKDCPKSASGCDPCECLKCSRK<br>GCECDCCPQKNDCCEAFCQGCKNCPPGCNPCKCTLNFCAKICNECKDCPKSDIGCDPCNCVKCSAKGCKCDCCPKKCC
OdiMT2$_{CAT}$	MH577044	MEVKRPNNCCPAKCLGCKGCPPGCEPCICKMDTCKNICNKCKECPKNEFGCDPCKCPKCSKLGCTCDCCHKKCCVTDCDG<br>SKTCPPGCEPCKCSMNACKKVCKQCKNCRKSESGCDPCECSKCALKGCKCDCCPKDTCCEVSCEGCKNCPPGCEPCKCTM<br>NSCMKTCDKCKDCPKSASGCDPCECRKCSRKGCDCDCCPQKNDSCEVFCQGCKNCPPGCNPCKCTLNFCAKICNECKDCP<br>KSDTGCDPCYCVKCSAKGCKCDCCPKKCS
ΨOdiMT2$_{CAT}$	MH577045	MEVKRLNNCCPAKCLGCKRCPPGCEPCICKMDTCKNICNKCARKTSSAATLASVLSAPSLDAHAIAATKNVVLPIAMDVR<br>LAPRAVNHASAQ
OdiMT2$_{ORE}$	MH577046	MEVKRPNNCCPAKCLGCKGCPPACDPCICKMDSCKNICNNCKECPKDESGCDPCECPKCAKLGCTCACCHKKCCIAACDG<br>CKTCPPGCEPCKCSMNACKKVCKQCKNCRKSESGCDPCECSKCASKGCKCDCCPKDNCCEASCEGCKNCPPGCEPCKCTM<br>NGCMKTCDKCKDCPKSENGCDPCNCRKCSRKGCDCDCCPSDDCCKASCEGCTNCPPGCDPCECSMDACKKSCEKCKDCR<br>KSESGCDPCECRKCSRKGCDCDCCPADSCCDASCDGCKNCPPGCEPCKCTMNGCMKTCDKCKDCPKSDSGCDPCECRKC<br>SRKGCDCDCCPSDSCCEASCRGCKNCPPGCEPCKCTLNCCMKTCEKCKDCPKSASGCDPCKCGKCSKKGCDCDCCPKKND<br>CCEAFCQGCKNCPPGCYPCKCTLNCCTKMCNECKNCPKSDTGCDPCKCAKCSTKGCKCDCCPKKCC
OdiMT2$_{OKI}$	SRA Project	MEVQQQRNNCCPAKCDGCKDCPPGCVPCKCLTDACTKICNNCKNCPPGCDPCGCEKCAKNGCTCDCCEQPCCETSCEG<br>CKNCPPGCNPCKCEMEKCKKICKECKTCPKGENGCDPCQCQKCSKMGCKCSCCPKQPCCEVSCAGCKNCPPGCNPCKCE<br>MASCKKICSGCADCPPGCDPCKCAKCSTKGCKCDCCPKKSCC
OdiMT1$_{OSA}$	SRA Project	MEVKRPNNCCIAACDGCKNCPPGCDPCKCSMKACKKVCEQCKNCRKSELGCDPCECSKCASEGCKCDCCPKDSCCEASCE<br>GCKDCPPGCEPCKCTMNGCMKICDKCKDCPKSENGCDPCNCRKCSRKGCDCDCCPSDDCCKASCEGCINCPPGCDPCECS<br>MDACKKSCEKCKDCRKSESGCDPCECRKCSRKGCDCDCCPADSCCEASCEGCKNCPPGCDPCKCTLNGCMKTCDKCKDCP<br>KSDKGCDPCQCKKCSRRGCECDCCPKKNDCCEAFCQGCKNCPPECNPCKCTLNCCTKMCNECKNCPKSDTGCDPCKCAK<br>CSTNGCKCDCCPKKCC
